# Hepatitis B virus X protein promotes vimentin expression via LIM and SH3 domain protein 1 to facilitate epithelial-mesenchymal transition and hepatocarcinogenesis

**DOI:** 10.1186/s12964-021-00714-1

**Published:** 2021-03-15

**Authors:** Hongjuan You, Dongchen Yuan, Yanwei Bi, Ning Zhang, Qi Li, Tao Tu, Xiao Wei, Qi Lian, Tong Yu, Delong Kong, Xiaoying Yang, Xiangye Liu, Xiaomei Liu, Fanyun Kong, Kuiyang Zheng, Renxian Tang

**Affiliations:** 1 Jiangsu Key Laboratory of Immunity and Metabolism, Department of Pathogenic Biology and Immunology, Xuzhou Medical University, Xuzhou, Jiangsu People’s Republic of China; 2 Clinical Laboratory, Xuzhou TCM Hospital Affiliated to Nanjing University of Chinese Medicine, Xuzhou, Jiangsu People’s Republic of China; 3 National Demonstration Center for Experimental Basic Medical Sciences Education, Xuzhou Medical University, Xuzhou, Jiangsu People’s Republic of China

**Keywords:** Hepatocellular carcinoma, HBX, Vimentin, LASP1, Epithelial-mesenchymal transition

## Abstract

**Background:**

Hepatitis B virus (HBV) X protein (HBX) has been reported to be responsible for the epithelial-mesenchymal transition (EMT) in HBV-related hepatocellular carcinoma (HCC). Vimentin is an EMT-related molecular marker. However, the importance of vimentin in the pathogenesis of HCC mediated by HBX has not been well determined.

**Methods:**

The expression of vimentin induced by HBX, and the role of LIM and SH3 domain protein 1 (LASP1) in HBX-induced vimentin expression in hepatoma cells were examined by western blot and immunohistochemistry analysis. Both the signal pathways involved in the expression of vimentin, the interaction of HBX with vimentin and LASP1, and the stability of vimentin mediated by LASP1 in HBX-positive cells were assessed by western blot, Co-immunoprecipitation, and GST-pull down assay. The role of vimentin in EMT, proliferation, and migration of HCC cells mediated by HBX and LASP1 were explored with western blot, CCK-8 assay, plate clone formation assay, transwell assay, and wound healing assay.

**Results:**

Vimentin expression was increased in both HBX-positive hepatoma cells and HBV-related HCC tissues, and the expression of vimentin was correlated with HBX in HBV-related HCC tissues. Functionally, vimentin was contributed to the EMT, proliferation, and migration of hepatoma cells mediated by HBX. The mechanistic analysis suggested that HBX was able to enhance the expression of vimentin through LASP1. On the one hand, PI3-K, ERK, and STAT3 signal pathways were involved in the upregulation of vimentin mediated by LASP1 in HBX-positive hepatoma cells. On the other hand, HBX could directly interact with vimentin and LASP1, and dependent on LASP1, HBX was capable of promoting the stability of vimentin via protecting it from ubiquitination mediated protein degradation. Besides these, vimentin was involved in the growth and migration of hepatoma cells mediated by LASP1 in HBX-positive hepatoma cells.

**Conclusion:**

Taken together, these findings demonstrate that, dependent on LASP1, vimentin is crucial for HBX-mediated EMT and hepatocarcinogenesis, and may serve as a potential target for HBV-related HCC treatment.

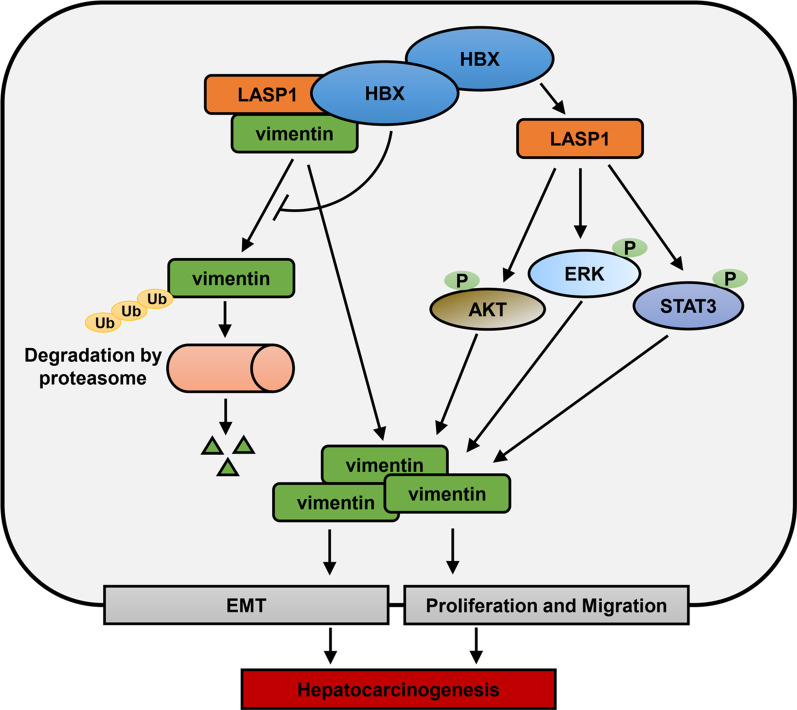

**Video abstract**

**Supplementary Information:**

The online version contains supplementary material available at 10.1186/s12964-021-00714-1.

## Background

Until now, hepatocellular carcinoma (HCC) is still one of the most important causes of cancer-related death globally [1, 2]. Despite several progresses in clinical diagnosis and treatment of the tumor that have been achieved, the prognosis of HCC remains poor, owing to the high rate of recurrence or metastasis. Among all of the etiologies, chronic hepatitis B virus (HBV) infection is a major pathogenic factor for the development of HCC [3, 4]. HBV X protein (HBX), a multifunctional protein encoded by the virus, is considered to play significant roles in HBV-induced hepatocarcinogenesis [5]. Although extensive studies have expanded our understanding of the functions of HBX in the pathogenesis of HCC [6], the molecular mechanisms associated with the progression of the tumor mediated by the viral protein are still not well clarified.

Epithelial-mesenchymal transition (EMT) is a significant event in the development of HBV-associated HCC [7]. HBX has been reported to induce the EMT phenotype in hepatoma cells [8–11], with the decreased levels of epithelial cell–cell adhesion molecule E-cadherin, while the increased expressions of cytoskeletal actin component β-catenin and vimentin. Moreover, HBX can regulate the expression of E-cadherin and β-catenin with multiple molecular mechanisms [11]. For example, HBX has been demonstrated to repress E-cadherin expression via histone deacetylation of E-cadherin through recruitment of the HDAC1 complex to the E-cadherin promoter [12], methylation of the E-cadherin promoter via DNMT1 activation [13]. In addition, HBX contributes to the increase of β-catenin by stabilizing β-catenin via GSK3β suppression [14], attenuating the interaction with of β-catenin with SIRT1 [15], and activating the β-catenin promoter through URG11 [16]. As a mesenchymal marker, vimentin plays a very important role in EMT [17]. However, whether vimentin participates in the dysfunction of hepatoma cells mediated HBX, and the molecular mechanisms associated with vimentin expression mediated by HBX are not clear.

LIM and SH3 domain protein 1 (LASP1) is a cytoskeleton protein that has been identified to participate in the development of human cancers with several types [18, 19], and facilitate the growth and invasion of glioma cells and colorectal cancer cells via EMT [20, 21]. The upregulation of LASP1 is also observed in HCC tissues, and its expression is closely related to HBV infection [22]. Moreover, we previously demonstrated that HBX could promote LASP1 expression via the activation of c-Jun [23, 24]. Except these, LASP1 has been reported to be capable of interacting with vimentin in hepatoma cells [25]. However, whether LASP1 is associated with an increase of vimentin mediated by HBX to facilitate EMT-associated hepatocarcinogenesis is unknown. In the present study, we explored the impact of LASP1 on vimentin expression mediated by HBX. Besides, the biological functions and potential molecular mechanisms associated with vimentin mediated by HBX via LASP1 in hepatoma cells was also investigated. Our findings could help us better understand the molecular mechanisms related to the development of HBV-related HCC regulated by HBX.

## Materials and methods

### Reagents and plasmids

The antibodies against HBX, LASP1, vimentin, E-cadherin, β-catenin, β-actin, AKT, Phosphorylated AKT (p-AKT, S473), ERK, Phosphorylated ERK (p-ERK, Thr202/Tyr204), STAT3, phosphorylated STAT3 (p-STAT3, Y705), P65, phosphorylated P65 (p-P65, Ser276), GAPDH, and HRP-conjugated secondary antibodies were obtained as mentioned previously [24, 26, 27]. The antibodies against Flag and HA Tags were from Abmart (Shanghai, China). PI3-K pathway inhibitor LY294002, ERK pathway inhibitor U0126, STAT3 pathway inhibitor NSC74859, NF-κB pathways inhibitor BAY11-7082, were purchased from Beyotime (Shanghai, China). Trizol reagent, G418, Matrigel solution, LipoMax DNA transfection reagent, and Clarity™ ECL western blot substrate were collected as previously described [24, 26]. Cycloheximide (CHX) and MG132 were from MCE (Medchem Express, Shanghai, China). Nuclear and Cytoplasmic Protein Extraction Kit was from Beyotime (Shanghai, China). HBX plasmids, and the plasmid containing short hairpin RNA (shRNA) against LASP1 were obtained as mentioned previously [23]. A plasmid containing shRNA against vimentin (GTACGTCAGCAATATGAAA), as well as a control plasmid, were purchased from GenePharma (Suzhou, Jiangsu, China).

### Cell culture and transfection

The culture for HepG2, Huh7, and HEK293T cells was followed as described [26–28]. According to the manual, the cells were transfected with target plasmids with LipoMax DNA transfection reagent. In addition, HepG2 and Huh7 cells stably transfected with target plasmids were selected with G418.

### Plasmids construction

The genes of LASP1 and vimentin were amplified by polymerase chain reaction (PCR) and cloned into a pcDNA3.1-Flag vector and pcDNA3.1-HA vector to construct the LASP1 (LASP1-Flag) and vimentin (vimentin-Flag and vimentin-HA) plasmids. The primer sequences for the LASP1 and vimentin genes were as follows: GTAGCTAGCGCCAGTTCCCCAGCTCCAG and TCCGCAGGTAAAAATTATACTTTTATTTGCG, GGGTACCGGGAGGCCCACGTATGGC and CGGAATTCTAGGAGTTTTTCCAAAGATTTATTGAAGCA. HBX plasmid was constructed relied on pcDNA3.1-Flag and pcDNA3.1-HA plasmid, three HBX mutant plasmids were constructed by using pcDNA3.1-HA plasmid, the LASP1 mutant plasmids were constructed based on pcDNA3.1-Flag plasmid, and the vimentin mutant plasmids were constructed dependent on pcDNA3.1-HA plasmid. The sequences of primers used for the HBX and associated mutants were followed previously [24]. The sequences of primers used for the LASP1 mutants were: LASP1-1: GGGGTACCAACCCCAACTGCGCCCGG and GCTCTAGAGCCACCACCTGGGGCGCT; LASP1-2: GGGGTACCCAAGCAGTCCTTCAC CATGGTGGC and GCTCTAGAGCCACCACCTGGGGCGCTG; LASP1-3: GGGGTACCCAAGCAGTCCTTCACCATGGTG and GCTCTA GATCAGATGGCCTCCACGTAGTTG. The sequences of primers used for the vimentin mutants were: vimentin-1: GGGGTACCTCCACCAGGTCCGTGTCC and GCTCTAGACTCCTCGCCTTCCAGCAG; vimentin-2: GGGGTACCCAAGAACACCCGCACCAACG and GCTCTAGACTCCTCGCCTTCCAGCAGCTT; vimentin-3: GGGGTACCCAAGAACACCCGCACCAA and GCTCTAGATTATTCAAGGT CATCGTGATGC. These expression vectors were verified by sequencing. The conditions for amplification were as follows: 2 min at 94 °C followed by 30 s at 94 °C, 45 s at 60 °C or 62 °C, and 45 s at 72 °C for 45 cycles.

### Clinical samples

The adjacent tissues (n = 60), as well as HCC tissues with HBV infection (n = 100) were collected from the Department of Pathology, Affiliated hospital of Xuzhou Medical University, or Outdo Biotech Co., Ltd (Shanghai, China). The study was followed by the principles of the Declaration of Helsinki. Approval was obtained from the ethics committee of Xuzhou Medical University. In addition, written informed consent was obtained from the patients.

### Western blot analysis

Western blot was performed as previously described [26]. Briefly, the total proteins were subjected to sodium dodecyl sulfate–polyacrylamide gel electrophoresis. Next, the target proteins were transferred onto polyvinylidene difluoride (PVDF) membranes. After blocking for 2 h with 5% milk in Tris-buffered saline, the PVDF membranes were incubated with target primary antibodies at 4 °C overnight and incubated with HRP-conjugated secondary antibodies for 2 h at the room temperature in the next day. The bands were detected by Clarity™ ECL western blot substrate.

### Immunohistochemistry (IHC) analysis

The IHC was performed, and the results were observed, as followed as described by Kong et al. [26]. Briefly, the fixed tissues with 4% formaldehyde, were embedded in paraffin. Next, the tissue sections were deparaffinized, rehydrated, and incubated with 0.01 M sodium citrate. After treated with 3% H_2_O_2_, and blocked with 5% goat serum, the tissue sections were incubated with LASP1, vimentin, or HBX antibodies overnight, and incubated with HRP-conjugated antibodies were for 2 h. Then, the tissue sections were stained by 3,3′-diaminobenzidine, terminated with double-distilled water, and further counterstained with hematoxylin. The expressions of LASP1, vimentin, and HBX in target tissues were calculated as described previously [26]. Briefly, to evaluate the expression levels of target proteins, the intensity of staining of hepatoma cells was scored as below: 0, no staining; 1, weak staining; 2: moderate staining; and 3, strong staining. The intensity score was < 2 was treated as low expression, and the intensity score was ≥ 2 was regarded as high expression.

### Immunofluorescence analysis

The locations of LASP1, vimentin, E-cadherin, β-catenin, and HBX proteins were measured with immunofluorescence assays as described [24]. Briefly, the target hepatoma cells were seeded on coverslips in 12-well plates. Then, the coverslips were fixed with ice-cold acetone, blocked with 5% bovine serum albumin in phosphate-buffered saline for 30 min, and incubated with different primary antibodies for 2 h. After washed with phosphate-buffered saline, the coverslips were incubated with Alexa Fluor 488, Alexa Fluor 647, and AMCA-conjugated secondary antibodies for 2 h. Finally, the results were observed by an Olympus microscope.

### Animal transplantation

The animal experiments were approved by the Animal Care and Use Committee of Xuzhou Medical University. Female BALB/c nude mice were purchased from Shanghai Laboratory Animal Co., Ltd., and fed under specific-pathogen-free, temperature-controlled conditions. After HepG2-HBX cells transfected with vimentin shRNA or control plasmids for 48 h, the target cells were resuspended in phosphate-buffered saline with the concentration of 1 × 10^7^/ml. Next, 0.1 ml target cell suspensions with 0.1 ml Matrigel solution were injected into the shoulder of the null mice. After fed for 2 weeks, the null mice were killed and tumors were excised. The length and width of each tumor were measured as previously described [27].

### The assays associated with cellular proliferation and migration

CCK-8 assay, plate clone formation assay, transwell assay, and wound healing assay were conducted as previously described [27].

### Co-immunoprecipitation (Co-IP) assay

The Co-IP assay was performed as previously described [24]. Briefly, After the protein extracts from the target cells were collected, it was incubated with different primary antibodies, and Protein G Sepharose beads for 12 h at 4 °C. Immunoglobulin G (IgG) as a negative control. After the immunoprecipitates were washed 4 times, the target proteins were analyzed by western blot.

### GST-pull down assay

GST-HBX or GST-LASP1 was expressed in *E. coli* strain DH5α and bound to glutathione-Sepharose beads to purify. His-vimentin or His-LASP1 was also expressed in *E. coli* strain DH5α and purified by His-beads. His-vimentin or His-LASP1 was incubated with GST alone, GST-HBX, or GST-LASP1 bound to glutathione-Sepharose beads at 4 °C. The beads were precipitated, washed 3 times with binding buffer, and subjected to SDS-PAGE, and further analyzed by western blot.

### Ubiquitination assay

The target cells were lysed after treated with a proteasome inhibitor MG132 for 6 h, and the protein extracts were then mixed with ubiquitin antibodies. Then the Sepharose beads of protein A/G were added to the protein extracts and rotated gently for overnight at 4 °C. The beads were next collected and washed. The SDS-loading buffer was used to elute the immunoprecipitated proteins at 95 °C for 5 min, and western blot was applied for the analysis of the ubiquitination of target proteins.

### Statistical analysis

The data were presented as the means ± standard deviation (SD). The statistical analysis was performed with a *t* test or one-way ANOVA. The chi-square test was used to analyze the relative expression of LASP1, vimentin, and HBX proteins measured by IHC analysis, and determine the significance of correlations between different proteins. The semiquantitative western blot analysis were determined with ImageJ software (NIH, Bethesda, MD, USA). A *p* value < 0.05 was statistically significant.

## Results

### HBX upregulates vimentin protein expression to facilitate EMT of hepatoma cells

**Fig. 1:**
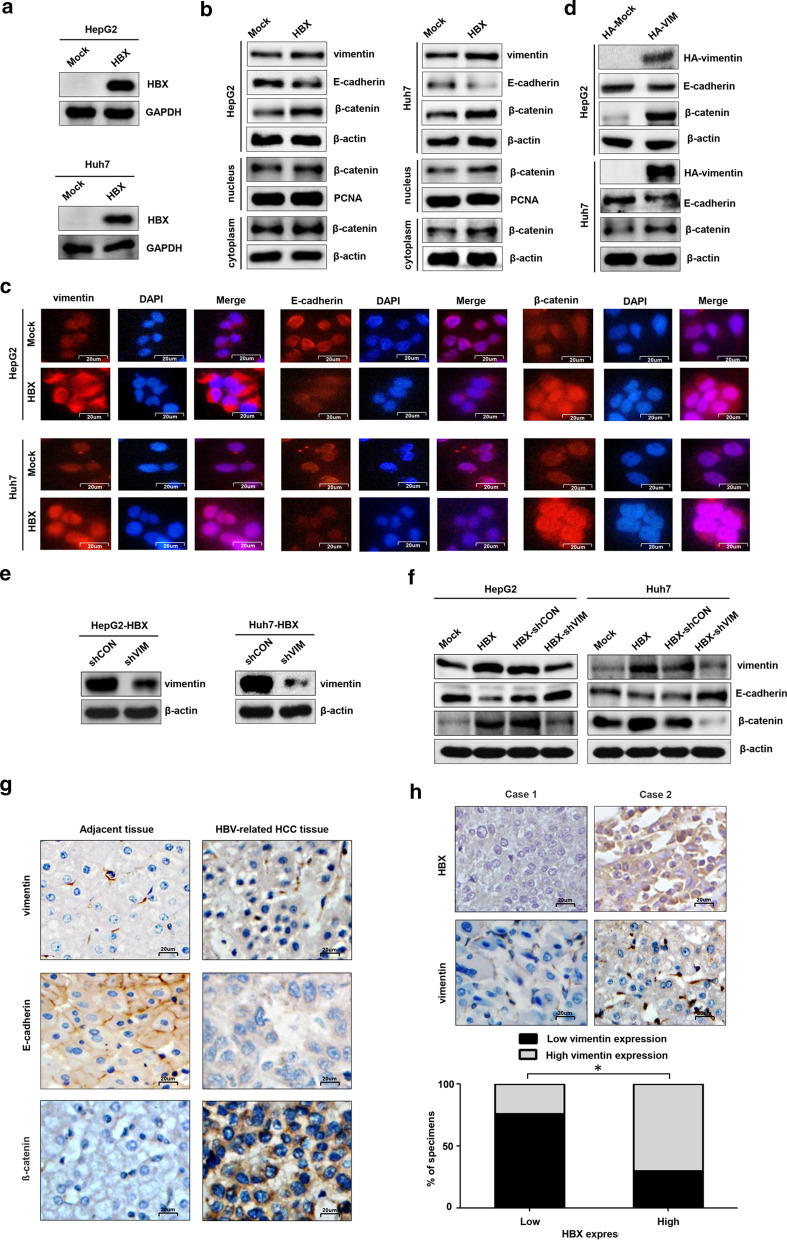
HBX promotes EMT via vimentin. **a** The stable expression of HBX in hepatoma cells was detected by western blot. **b** The effect of HBX on expressions of EMT markers, including vimentin, E-cadherin, and β-catenin in hepatoma cells measured by western blot. **c** The effect of HBX on expressions of EMT markers, including vimentin, E-cadherin, and β-catenin in hepatoma cells were detected by immunofluorescence assay (× 400). **d** The role of over-expressed vimentin on E-cadherin and β-catenin in hepatoma cells. **e** The effect of shRNA on the expression of vimentin protein. **f** The inhibition of vimentin on the E-cadherin and β-catenin expressions in HBX-positive hepatoma cells. **g** The expressions of vimentin, E-cadherin, and β-catenin in HBV-related HCC tissues (n = 100) and associated adjacent tissues (n = 60) (× 400). **h** The correlation between HBX and vimentin protein expression in HBV-related HCC tissues. Mock: cells transfected with control plasmid. HBX: cells transfected with HBX plasmid. HA-Mock: cells transfected with control expression plasmid with HA tags. HA-VIM: cells transfected with vimentin expression plasmid with HA tags. HBX-shCON: HBX-positive cells transfected with shRNA control plasmid. HBX-shVIM: HBX-positive cells transfected with shRNA plasmid targeting vimentin. *P < 0.05

To explore the effect of HBX on EMT, we constructed the hepatoma cells stably transfected with HBX (Fig. 1a). EMT is a well-known critical event in the progression of HCC [7, 10], and we detected the expressions of EMT markers, including vimentin, E-cadherin, and β-catenin proteins in HBX-positive hepatoma cells and control cells. The results showed that HBX could promote EMT by increasing the expressions of vimentin and β-catenin and declining E-cadherin expression (Fig. 1b). During EMT, β-catenin is released from the cellular membrane to enter the cytoplasmic pool, and further translocated into the nucleus [11]. In the present study, the expression of β-catenin in cytoplasm and nucleus mediated by HBX was explored by western blot. The results showed that the expression of β-catenin in cytoplasm and nucleus was upregulated in HBX-positive hepatoma cells (Fig. 1b). We also measured the expression of vimentin, E-cadherin, and β-catenin proteins in hepatoma cells by immunofluorescence assay. Compared to control cells, the increased expression of vimentin and β-catenin, and decreased E-cadherin were observed in HBX-positive hepatoma cells. The increased expression of β-catenin in cytoplasm and nucleus was also found in HBX-positive hepatoma cells by immunofluorescence assay (Fig. 1c). As a mesenchymal marker, vimentin had a vital role in the EMT process [17]. We were interested in investigating whether HBX could promote EMT via vimentin, and the effect of vimentin on the expressions of EMT markers, including E-cadherin and β-catenin, in hepatoma cells. As shown in Fig. 1d, we found that the expression of E-cadherin was declined, but β-catenin was enhanced in vimentin-overexpressed hepatoma cells. Furthermore, vimentin-specific shRNA was used to knock down the expression of vimentin protein in HBX-positive hepatoma cells (Fig. 1e). We found that, when the suppression of vimentin, the E-cadherin expression was increased, while β-catenin expression was reduced in HBX-positive hepatoma cells (Fig. 1f).

We also explored the expressions of vimentin, E-cadherin, and β-catenin in HBV-related HCC tissues and associated adjacent tissues by IHC analysis. The results showed that, compared to adjacent tissues, increased vimentin, β-catenin, and decreased E-cadherin expressions were found in HBV-associated HCC tissues (Fig. 1g). Moreover, we found that the expression of vimentin protein was associated with HBX in HBV-related tumor tissues (Fig. 1h). Taken together, these results indicated that HBX could enhance vimentin expression to facilitate EMT in hepatoma cells.

### Vimentin induced by HBX promotes the proliferation and migration of hepatoma cells

**Fig. 2:**
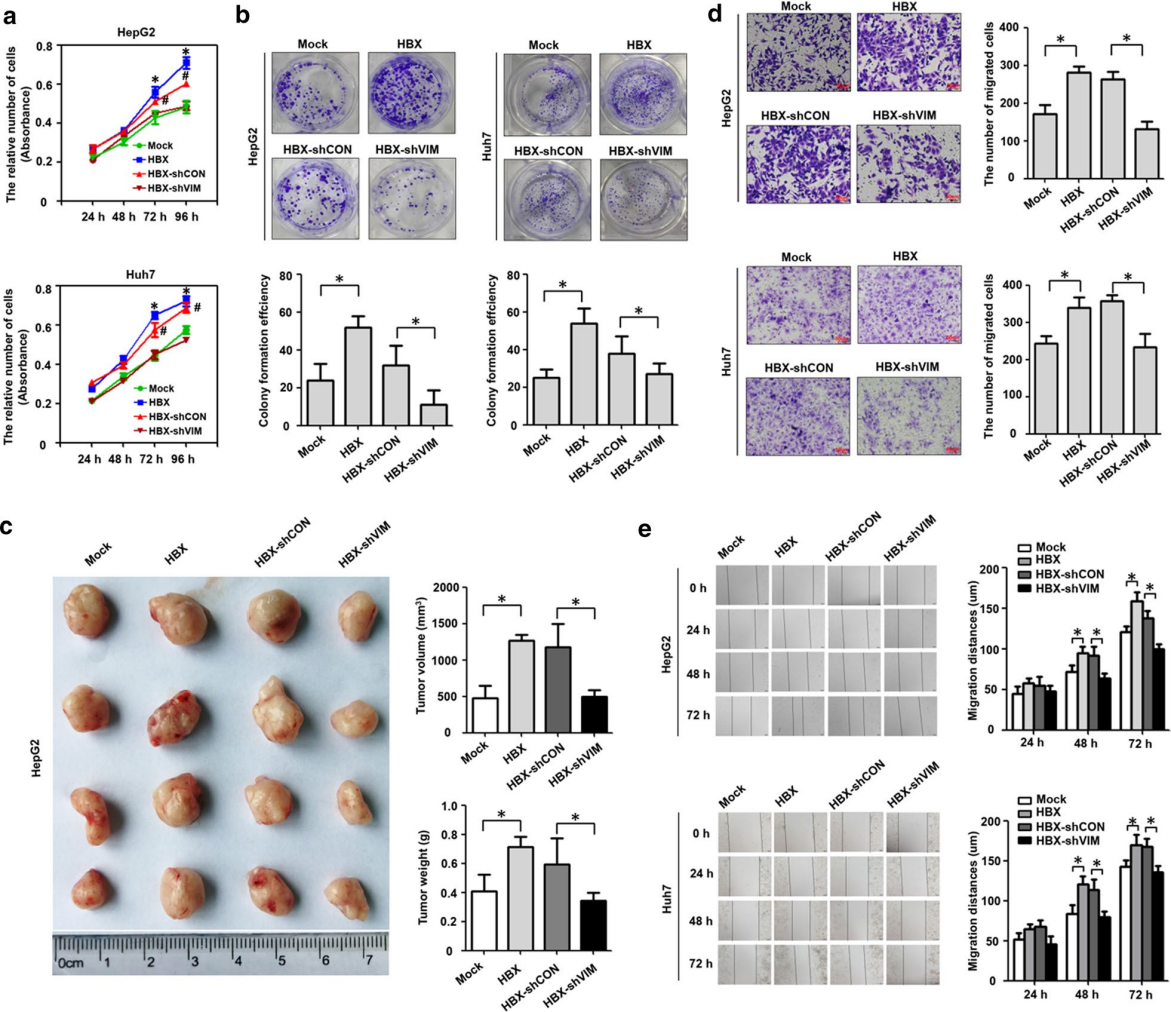
HBX promotes the growth and migration of hepatoma cells through vimentin. **a** The effect of inhibition of vimentin expression using shRNA on the proliferation of HBX-positive hepatoma cells detected with CCK-8 assays. **b** The effect of inhibition of vimentin expression via shRNA on the proliferation of HBX-positive hepatoma cells assessed with plate clone formation assays. **c** The role of vimentin in the proliferation of hepatoma cells mediated by HBX in nude mice. **d** The effect of inhibition of vimentin expression via shRNA on the migration of HBX-positive hepatoma cells detected with transwell assays. **e** The effect of inhibition of vimentin expression using shRNA on the migration of HBX-positive hepatoma cells examined with wound healing assays. Mock: cells transfected with control plasmid. HBX: cells transfected with HBX plasmid. HBX-shCON: HBX-positive cells transfected with shRNA control plasmid. HBX-shVIM: HBX-positive cells transfected with shRNA plasmid targeting vimentin. In **a**, *P < 0.05, the Mock group compared with the HBX group; ^#^*P* < 0.05, the HBX-shCON group compared with the HBX-shVIM group

Our published studies showed that HBX promotes the proliferation and migration of hepatoma cells [27], we next measured whether HBX could facilitate cellular proliferation and migration via vimentin. The results of cell viability and plate clonal formation assays showed that, compared to control cells, HBX-positive hepatoma cells exhibited higher proliferation efficiency (Fig. 2a, b). After HBX-positive hepatoma cells were treated with vimentin shRNA, the cellular proliferation mediated by HBX was reduced.

We also investigated the role of HBX and vimentin on the development of HCC in vivo. After HepG2-HBX cells (HepG2 cells with stable expression of HBX) were treated with vimentin shRNA, the cells were subcutaneously injected into nude mice. As the results presented in Fig. 2c, both the volume and the weight of the HepG2-HBX tumors were greater than those of control tumors. After HepG2-HBX cells transfected with shRNA against vimentin, the ability of HepG2-HBX cells to form tumors was significantly lower than that of cells treated with control shRNA in nude mice.

Next, transwell and wound healing assays were used to assess the effect of vimentin on cellular migration mediated by HBX. Our results indicated that HBX has capable of promoting the migration of hepatoma cells. When HBX-positive cells were treated with vimentin shRNA, the migration efficiency of hepatoma cells mediated by HBX was suppressed (Fig. 2d, e). Together, these findings suggested that vimentin was involved in the proliferation and migration mediated by HBX in hepatoma cells.

### LASP1 contributes to the expression of vimentin protein mediated by HBX in hepatoma cells

**Fig. 3:**
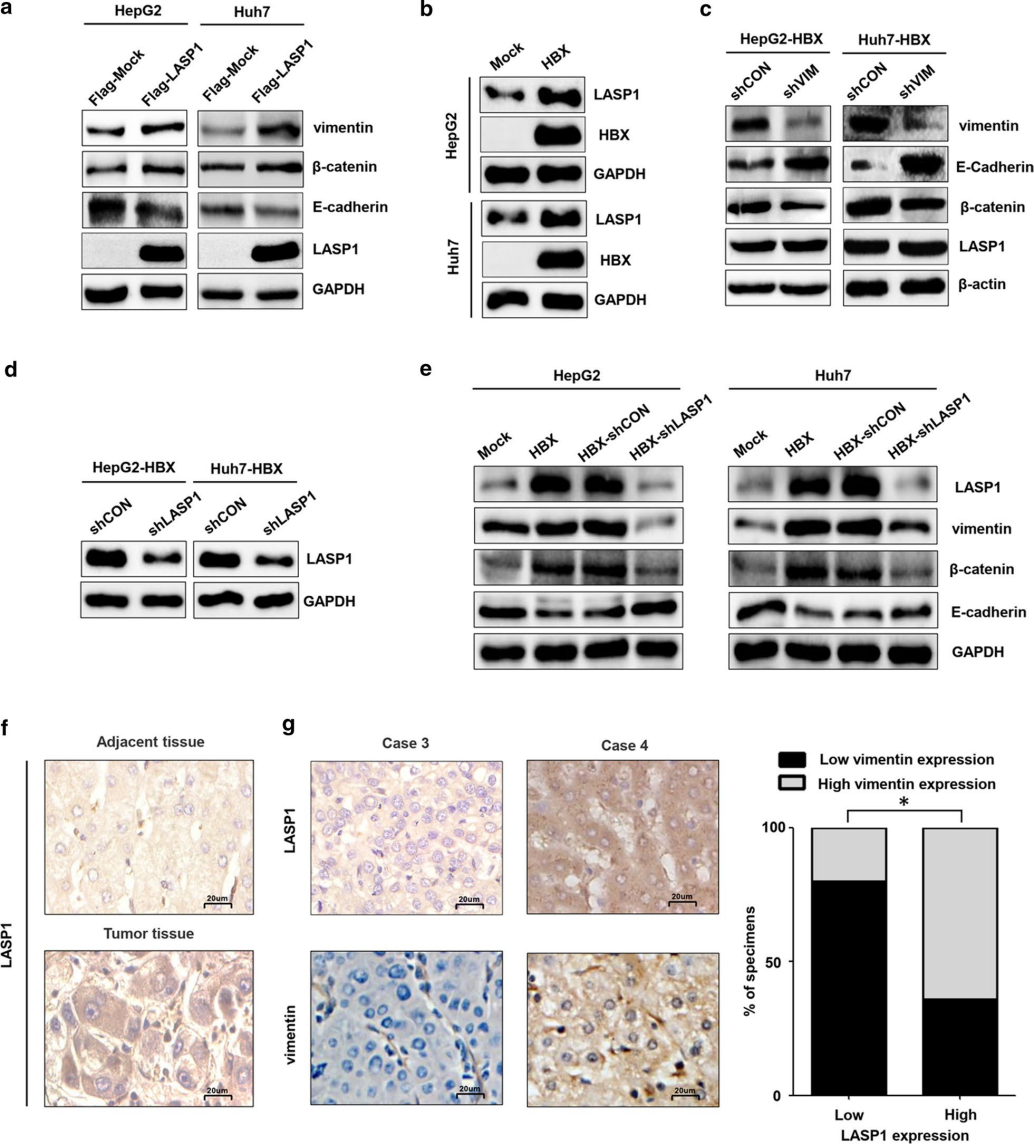
The effect of LASP1 on vimentin expression mediated by HBX in hepatoma cells. **a** The role of over-expressed LASP1 on the expressions of EMT markers, including vimentin, E-cadherin, and β-catenin in hepatoma cells. **b** The effect of HBX on the expression of LASP1 in hepatoma cells. **c** The inhibition of vimentin on the expression of LASP1 in HBX-positive hepatoma cells. **d** The inhibition of LASP1 mediated by specific shRNA in HBX-positive hepatoma cells. **e** The inhibition of LASP1 on the expression of vimentin, E-cadherin, and β-catenin in HBX-positive hepatoma cells. **f** The expression of LASP1 in HBV-related HCC tissues and associated adjacent tissues (× 400). **g** The correlation of LASP1 and vimentin protein expression in HBV-related HCC tissues (× 400). Flag-Mock: cells transfected with control expression plasmid with Flag tags. Flag-LASP1: cells transfected with LASP1 expression plasmid with Flag tags. Mock: cells transfected with control plasmid. HBX: cells transfected with HBX plasmid. shCON: cells transfected with shRNA control plasmid. shVIM: cells transfected with shRNA plasmid targeting vimentin. HBX-shCON: HBX-positive cells transfected with shRNA control plasmid. HBX-shLASP1: HBX-positive cells transfected with shRNA plasmid targeting LASP1. *P < 0.05

Our previous studies indicated that HBX could promote the expression of LASP1 in hepatoma cells [23, 24]. In addition, LASP1 was found to facilitate EMT in glioma and colorectal cancer cells [20], and interact with vimentin in HCC [25]. We examined whether LASP1 contributes to the vimentin expression mediated by HBX in hepatoma cells. At first, we investigated whether LASP1 could enhance EMT in hepatoma cells. As shown in Fig. 3a, we found that the expressions of vimentin and β-catenin were increased, but the expression of E-cadherin was declined in LASP1-overexpressed hepatoma cells. These results suggested that LASP1 was capable of promoting EMT in hepatoma cells. Furthermore, consistent with our previous researches, the expression of LASP1 was increased in HBX-positive cells (Fig. 3b). The suppression of vimentin has no significant role in LASP1 expression in HBX-positive hepatoma cells (Fig. 3c). However, after treated the HBX-positive cells with LASP1 shRNA (Fig. 3d, e), the expression of vimentin was inhibited. Meanwhile, the increased expression of E-cadherin and reduced expression of β-catenin were also found in HBX-positive and LASP1-inhibited hepatoma cells. These results suggested that LASP1 facilitated vimentin expression to promote EMT in HBX-positive hepatoma cells. We also explored the correlation of LASP1 and vimentin expression in HBV-related HCC tissues. Consistent with our published reports, compared with adjacent tissues, the expression of LASP1 was increased in HBV-related HCC tissues (Fig. 3f). Moreover, a significant correlation of LASP1 and vimentin expression was found in HBV-related HCC tissues (Fig. 3g). Taken together, these results indicated that HBX could promote the expression of vimentin through LASP1 in hepatoma cells.

### Multiple signal pathways are involved in the increase of vimentin induced by LASP1 in HBX-positive hepatoma cells

**Fig. 4:**
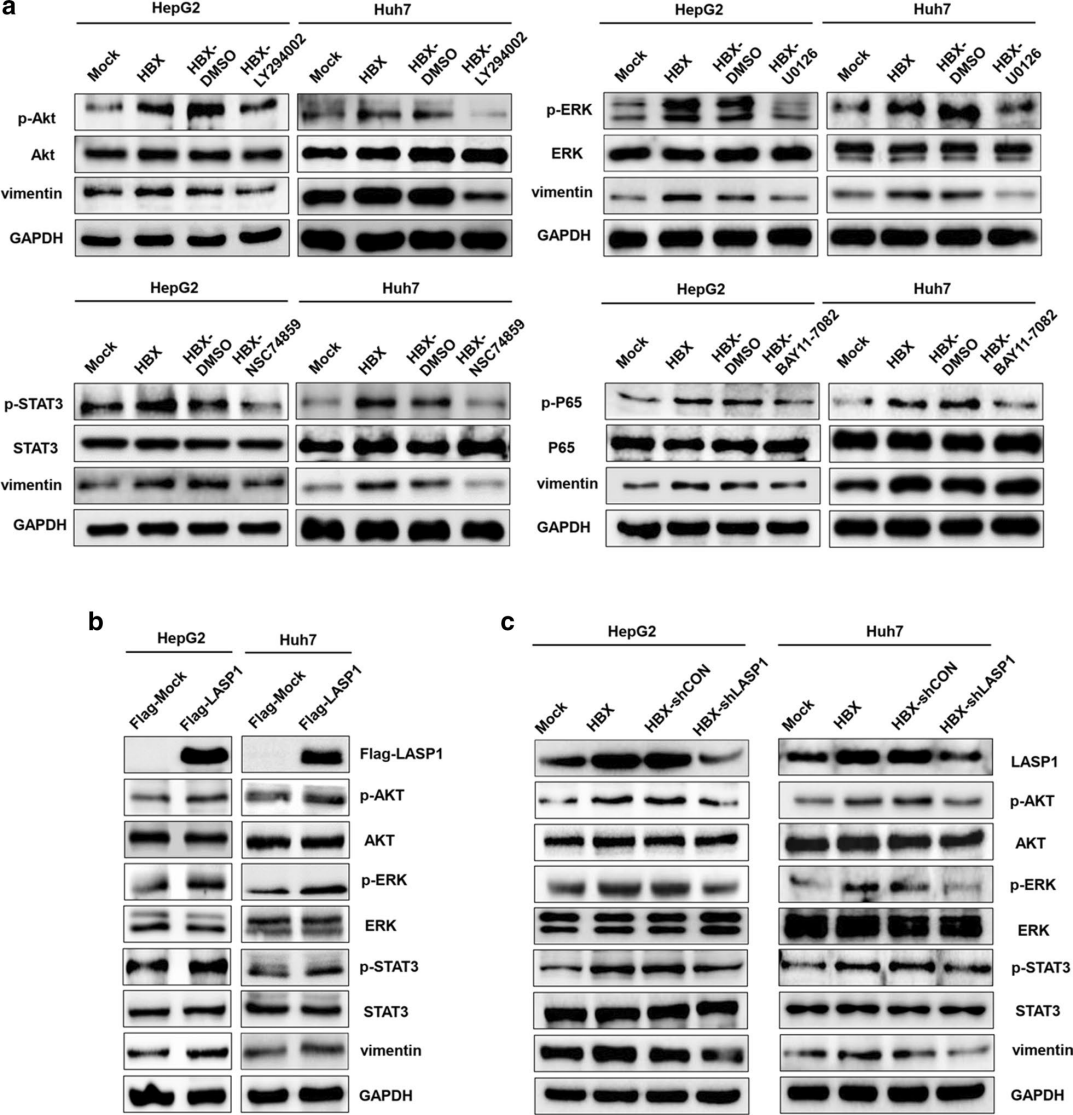
The signal pathways involved in the regulation of vimentin expression mediated by LASP1 in HBX-positive hepatoma cells. **a** The inhibition of PI3-K, ERK, STAT3, and NF-kB pathways on vimentin expression in HBX-positive hepatoma cells. **b** The effect of over-expressed LASP1 on activation of PI3-K, ERK, and STAT3 pathways in HBX-positive hepatoma cells. **c** The inhibition of LASP1 on activation of PI3-K, ERK, and STAT3 pathways and the expression of vimentin in HBX-positive hepatoma cells. Mock: cells transfected with control plasmid. HBX: cells transfected with HBX plasmid. Flag-Mock: cells transfected with control expression plasmid with Flag tags. Flag-LASP1: cells transfected with LASP1 expression plasmid with Flag tags. HBX-shCON: HBX-positive cells transfected with shRNA control plasmid. HBX-shLASP1: HBX-positive cells transfected with shRNA plasmid targeting LASP1

Next, we explored the mechanisms associated with the increase of vimentin mediated by LASP1 in HBX-positive hepatoma cells. Current reports showed that vimentin expression is mainly mediated by PI3-K [29], ERK [30], STAT3 [31], and NF-κB pathways in different cells [32]. We first examined whether HBX was able to promote vimentin expression via these pathways. The results showed that HBX could activate PI3-K, ERK, STAT3, and NF-κB pathways in hepatoma cells. When the cells were treated with the inhibitors of PI3-K, ERK, STAT3 pathways, the activities of AKT (a molecule in the PI3-K pathway), ERK, and STAT3 induced by HBX were abolished (Fig. 4a). Meanwhile, the vimentin expression was also suppressed. However, the expression of vimentin was unchanged in the cells treated with NF-κB pathway inhibitors. These results suggested that PI3-K, ERK, and STAT3 pathways contributed to the expression of vimentin in HBX-positive hepatoma cells.

Current studies showed that LASP1 could activate multiple pathway signals including PI3-K [20], ERK, and Smad2 pathways [21]. We investigated whether LASP1 could enhance vimentin expression via the signal pathways as mentioned above, and the results showed that over-expressed LASP1 could promote the activation of PI3-K, ERK, STAT3 pathways in hepatoma cells (Fig. 4b). Furthermore, after HBX-positive cells treated with LASP1 shRNA, the activation of PI3-K, ERK, STAT3 were suppressed. Meanwhile, vimentin expression was also decreased (Fig. 4c). Overall, these findings indicated that the activation of PI3-K, ERK, and STAT3 pathways mediated by LASP1 was responsible for vimentin protein expression in HBX-positive hepatoma cells.

### HBX is capable of interacting with LASP1 and vimentin in hepatoma cells

**Fig. 5:**
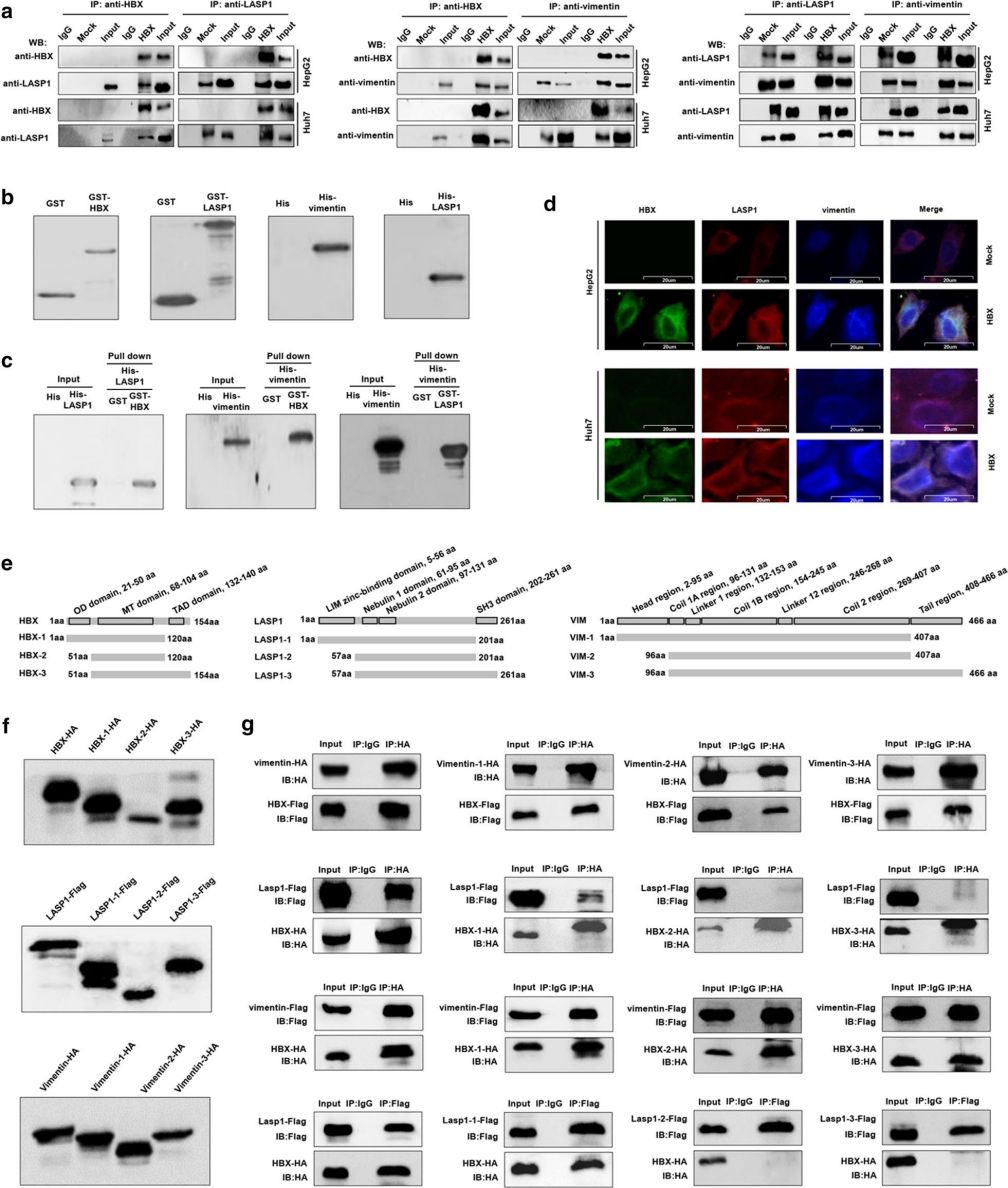
The interaction of HBX with LASP1 and vimentin and HBX promotes vimentin stability through LASP1 in hepatoma cells. **a** The interaction of HBX with LASP1 and vimentin measured by Co-IP assays in hepatoma cells. **b** The prokaryotic expressions of HBX with GST Tag, LASP1 with GST Tag, vimentin with His Tag, LASP1 with His Tag detected by western blot. **c** The interaction of HBX with LASP1 and vimentin measured by GST-pull down assays. **d** The colocalizations of HBX, LASP1, and vimentin in hepatoma cells were detected immunofluorescence assay (× 400). **e** The schematic diagram of three HBX mutants, LASP1 mutants, and vimentin mutants. The domain and region distribution in LASP1 and vimentin protein were as the description in the UniProt database. **f** The protein expressions of HBX and associated mutants with HA Tag, LASP1 and associated mutants with Flag Tag, vimentin and associated mutants with HA Tag, in HEK293T cells detected by western blot. **g** The interaction of HBX and associated mutants with LASP1, vimentin and associated with mutant in 293 T cells detected by Co-IP assay. *VIM* vimentin

Several studies have shown that HBX could mediate the dysfunction of hepatoma cells by interacting with different target proteins [33, 34]. We investigated whether HBX could interact with LASP1 and vimentin. As predicted, based on the Co-IP assay, we found that HBX could directly interact with LASP1 and vimentin (Fig. 5a). The prokaryotic expression plasmids of HBX, LASP1, and vimentin with different Tags were also constructed (Fig. 5b). Using GST-pull down assay, the interaction of HBX with LASP1 and vimentin in vitro was measured, and the results showed that HBX could directly interact with LASP1 and vimentin in vitro (Fig. 5c). In addition, consistent with the report from Salvi et al. [25], the interaction of LASP1 and vimentin was also found. Besides these, the co-location of HBX, LASP1, and vimentin was also found in hepatoma cells based on immunofluorescence assay (Fig. 5d).

To further characterize the region of HBX that is involved in the interaction with the regions of LASP1 and vimentin, three deletion mutants of the HBX, namely HBX-1 (1–120 aa, a C-terminal deletion mutant of HBX), HBX-2 (51–120 aa, a double-terminal deletion mutant of HBX), and HBX-3 (51–154 aa, an N terminal deletion mutant of HBX) was constructed based on domain distribution as mentioned previously [24] and Fig. 5e, f. Furthermore, based on the domain or region distribution in LASP1 and vimentin protein as described in UniProt database [35] and Fig. 5e, three deletion mutants of the LASP1, namely LASP1-1 (1–202 aa, a C-terminal deletion mutant of LASP1), LASP1-2 (57–202 aa, a double-terminal deletion mutant of LASP1), and LASP1-3 (57–261 aa, an N terminal deletion mutant of LASP1), and three deletion mutants of the vimentin, namely vimentin-1 (1–407 aa, a C-terminal deletion mutant of vimentin), vimentin-2 (96–407 aa, a double-terminal deletion mutant of vimentin), and vimentin-3 (96–466 aa, an N terminal deletion mutant of vimentin), were also constructed (Fig. 5e, f). The plasmids with different HBX, LASP1, and vimentin mutants were co-transfected into HEK293T cells, and the Co-IP results showed that the HBX-1 mutant could interact with LASP1, and the result suggested that the N-terminal of HBX, which had the OD domain, was responsible for the interacting with LASP1. HBX-1, HBX-2, and HBX-3 mutants could bind to vimentin, indicating the middle region of HBX protein, which had the MT domain, could bind with vimentin. Only the LASP1-3 mutant was capable of interacting with HBX, suggesting the C-terminal of LASP1 protein with the SH3 domain could interact with HBX. In addition, vimentin-1, vimentin-2, and vimentin-3 could bind to HBX, and these results indicated that the middle region of vimentin protein with multiple different regions could bind with HBX (Fig. 5g).

### LASP1 contributes to the stability of vimentin protein mediated by HBX in hepatoma cells

**Fig. 6:**
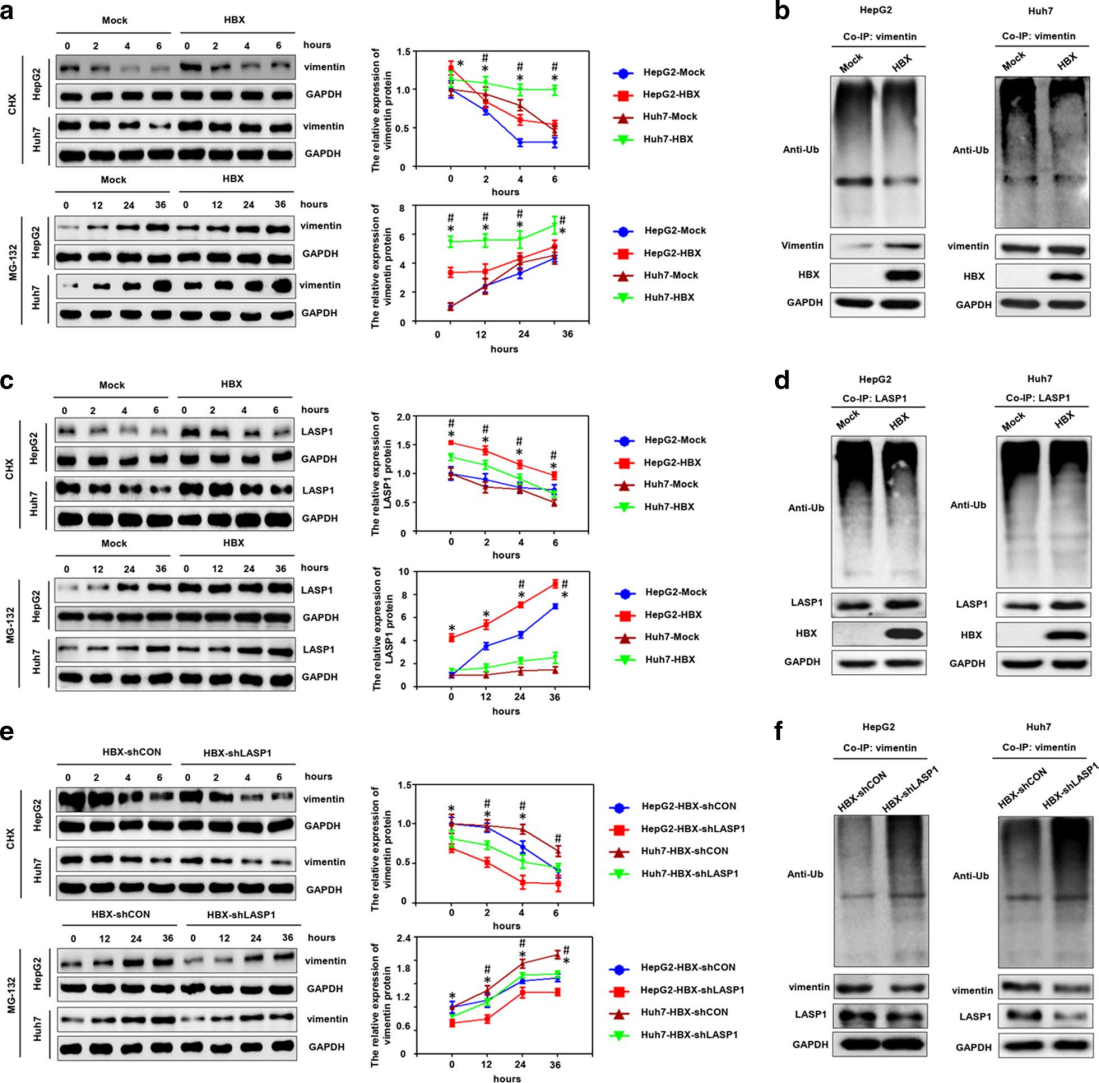
HBX enhances the stability of vimentin via LASP1. **a** HBX promoted vimentin stability in hepatoma cells. **b** HBX inhibited vimentin ubiquitination in hepatoma cells. **c** HBX promoted LASP1 stability in hepatoma cells. **d** HBX inhibited LASP1 ubiquitination in hepatoma cells. **e** HBX promoted vimentin stability through LASP1 in hepatoma cells. **f** HBX inhibited vimentin ubiquitination through LASP1 in hepatoma cells. Mock: cells transfected with control plasmid. HBX: cells transfected with HBX plasmid. HBX-shCON: HBX-positive cells transfected with shRNA control plasmid. HBX-shLASP1: HBX-positive cells transfected with shRNA plasmid targeting LASP1. *P < 0.05, HepG2-Mock compared to HepG2-HBX, or HepG2-HBX-shCON compared to HepG2-HBX-shLASP1; ^#^*P* < 0.05, Huh7-Mock compared to Huh7-HBX, or Huh7-HBX-shCON compared to Huh7-HBX-shLASP1

The ubiquitin–proteasome system (UPS) has been verified to play an essential role in regulating the stability of target proteins [36]. Since current studies have shown that HBX could significantly influence the stability of target proteins, which could bind to the viral protein, by regulating the UPS [37], we speculated that the degradation of vimentin by the UPS may be influenced by HBX. The stability of vimentin was determined after the hepatoma cells were treated with a protein synthesis inhibitor CHX. We found that HBX significantly increased the half-life of vimentin (Fig. 6a). In addition, the expression of vimentin was examined in hepatoma cells after treatment with a proteasome inhibitor MG132. The results showed that HBX has the capability of increasing the stability level of vimentin (Fig. 6a). Furthermore, HBX could reduce the ubiquitination level of vimentin in hepatoma cells (Fig. 6b). Because HBX also interacted with LASP1, we also investigated the effect of HBX on LASP1 stability in hepatoma cells in the present study. We found that HBX also could promote the stability of LASP1 and reduce the ubiquitination of LASP1 (Fig. 6c, d). As mentioned above, HBX could promote the expression of vimentin via LASP1, we next examined whether HBX could facilitate the stability of vimentin and inhibit the ubiquitination of vimentin through LASP1. As expected, the results showed that the stability level of vimentin was decreased, and the ubiquitination level of vimentin was enhanced, when HBX-positive hepatoma cells were incubated with LASP1 shRNA (Fig. 6e, f). Taken together, these results suggested that HBX could interact with vimentin and LASP1, and protect vimentin from ubiquitination and degradation via LASP1 in hepatoma cells.

### Vimentin is associated with the increased proliferation and migration induced by LASP1 in HBX-positive hepatoma cells

**Fig. 7:**
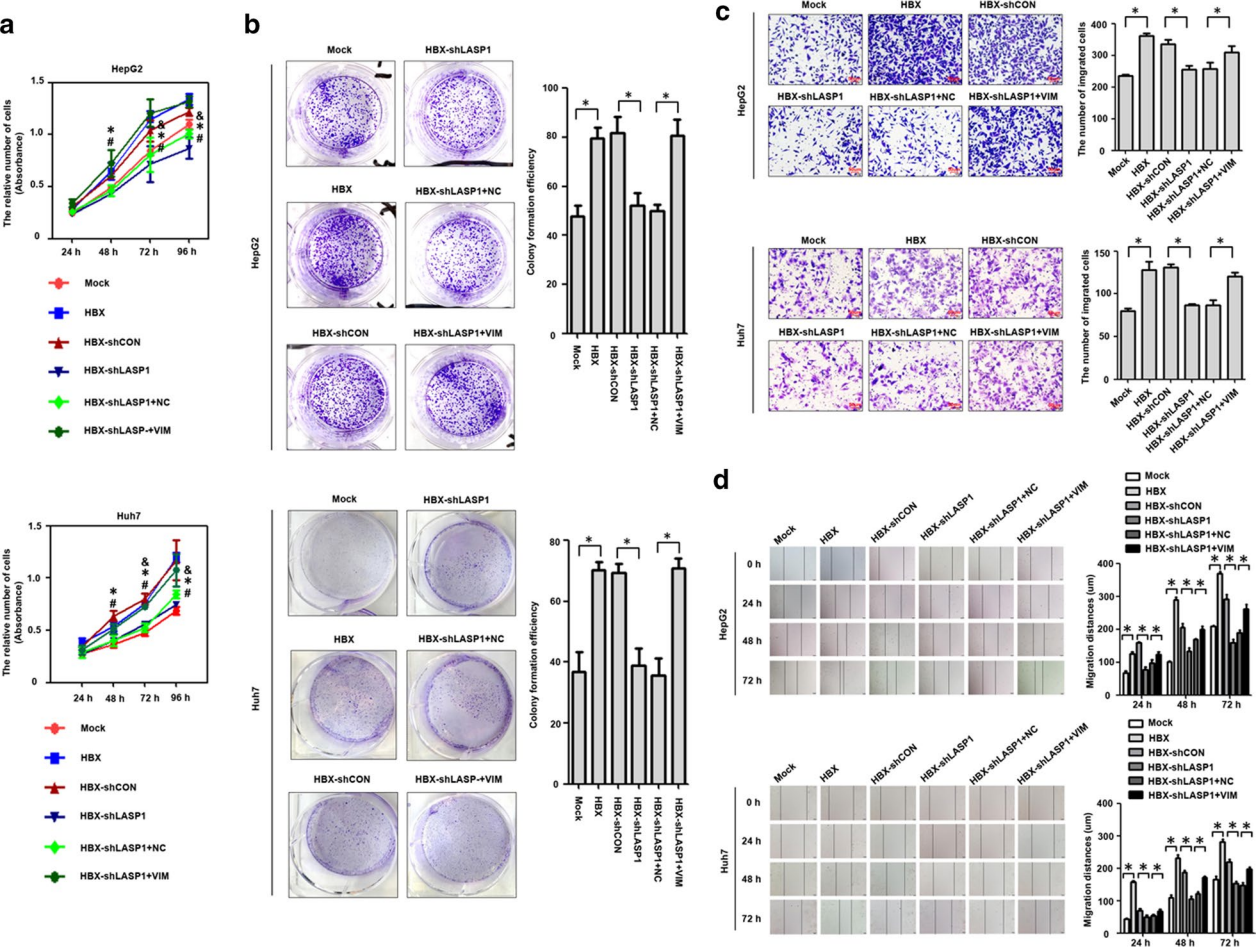
LASP1 enhances proliferation and migration of HBX-positive hepatoma cells via vimentin. **a** The effect of vimentin on the proliferation of HBX-positive hepatoma cells mediated by LASP1 was detected with CCK-8 assays. **b** The effect of vimentin on the proliferation of HBX-positive hepatoma cells mediated by LASP1 was assessed with plate clone formation assays. **c** The effect of vimentin on the migration of HBX-positive hepatoma cells mediated by LASP1 was detected with transwell assays. **d** The effect of vimentin on the migration of HBX-positive hepatoma cells mediated by LASP1 was assessed with wound healing assays. Mock: cells transfected with control plasmid. HBX: cells transfected with HBX plasmid. HBX-shCON: HBX-positive cells transfected with shRNA control plasmid. HBX-shLASP1: HBX-positive cells transfected with shRNA plasmid targeting LASP1. HBX-shLASP1 + NC: HBX-positive with shRNA plasmid targeting LASP1 cells transfected with control expression plasmids of vimentin. HBX-shLASP1 + NC: HBX-positive with shRNA plasmids targeting LASP1 cells transfected with vimentin expression plasmids. *P < 0.05, the Mock group compared with the HBX group; ^#^*P* < 0.05, the HBX-shCON group compared with the HBX-shLASP1 group. ^&^*P* < 0.05, the HBX-shCON + NC group compared with the HBX-shLASP1 + VIM group

We previously demonstrated that HBX could promote the proliferation and migration of hepatoma cells via LASP1 [23]. In this study, we were interested in measuring whether vimentin was involved in the cellular proliferation and migration mediated by LASP1 in HBX-positive cells. Consistent with our published researches, the results of cell viability and plate clonal formation assays showed that, after inhibition of LASP1 by shRNA, the HBX-positive cells exhibited lower proliferation efficiency than control cells (Fig. 7a, b). However, when the HBX-positive cells with decreased LASP1 were treated with vimentin expression vectors, the cellular proliferation ratio was increased.

Transwell and wound healing assays were next utilized to investigate the effect of vimentin in cell migration mediated by LASP1 in HBX-positive hepatoma cells. We found that LASP1 inhibition could reduce the migration ability of HBX-positive hepatoma cells. When HBX-positive cells with decreased LASP1 were treated with vimentin expression vectors, the migration capability of hepatoma cells was enhanced (Fig. 7c, d). Together, these results indicate that vimentin was involved in the proliferation and migration mediated by LASP1in HBX-positive hepatoma cells.

## Discussion

**Fig. 8:**
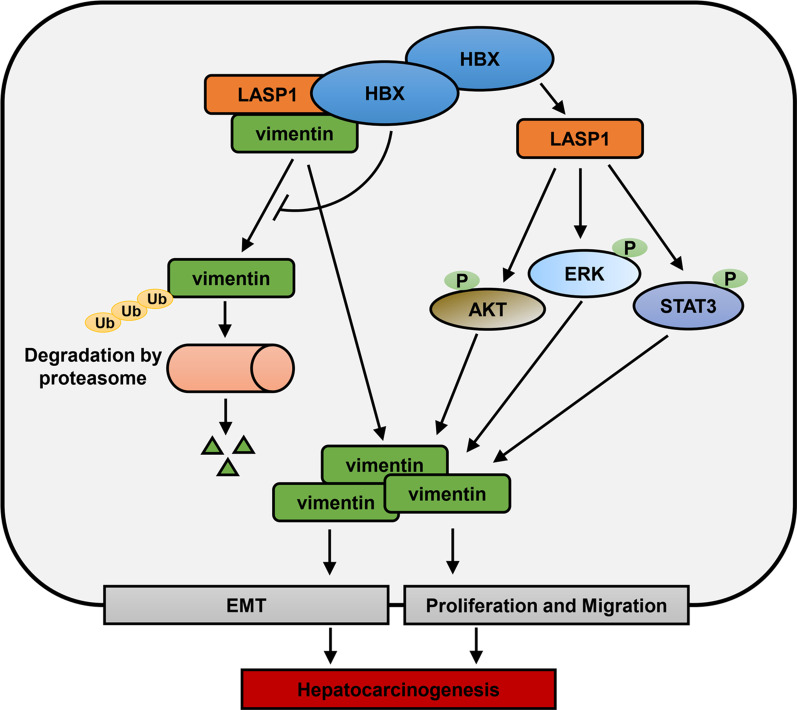
A schematic diagram showing the mechanisms associated with the increase of vimentin induced by HBX to facilitate hepatocarcinogenesis. HBX promotes vimentin expression in hepatoma cells to facilitate epithelial-mesenchymal transition, proliferation and migration, which is responsible for hepatocarcinogenesis. In addition, HBX could enhance vimentin expression through LASP1. On the one hand, multiple signal pathways, including PI3-K, EKR, and STAT3 are involved in the upregulation of vimentin mediated by LASP1 in HBX-positive hepatoma cells. On the other hand, HBX directly interacts with vimentin and LASP1, and dependent on LASP1, HBX could promote vimentin stability via protecting it from ubiquitination mediated protein degradation

Current studies have shown that EMT is involved in the hepatocarcinogenesis mediated by HBX [8–10]. As a mesenchymal marker, vimentin is essential for EMT [17]. However, the role and molecular mechanisms related to vimentin mediated by HBX are still unclear. In the present study, we demonstrated that HBX could facilitate vimentin expression via LASP1 to promote EMT, proliferation, and migration of hepatoma cells. Furthermore, different molecular mechanisms were involved in the expression of vimentin mediated by LASP1 in HBX-positive hepatoma cells (Fig. 8), and these findings may help us better understand the molecular mechanism of tumorigenesis mediated by HBX during HBV infection.

As a cancer cofactor, HBX has been verified to exhibit a vital effect on EMT in HCC [10], and the early reports indicated that the viral protein could regulate the expressions of two EMT markers, E-cadherin and β-catenin with different molecular mechanisms [11]. Vimentin also was involved in the EMT process mediated by HBX, while the associated molecular function and mechanisms have not been well clarified. In the present study, we found that vimentin could mediate the expressions of E-cadherin and β-catenin in HBX-positive hepatoma cells, and facilitate the growth and migration of hepatoma cells, indicating that vimentin plays a very important role in EMT and hepatocarcinogenesis mediated by HBX.

LASP1 overexpression has been reported in HBV-related HCC [22], and our previous works have indicated that HBX was responsible for the increase of this protein in liver cancer [23, 24]. However, the molecular mechanism associated with the development of HCC mediated by LASP1 in HBV-related HCC is still not well illustrated. Because current studies indicated that LASP1 had the capability of promoting EMT in glioma cells and colorectal cancer cells [20, 21], and the interaction of LASP1 and vimentin was also reported [25], in the present study, we were interested in examining whether LASP1 could induce EMT via vimentin in HCC mediated by HBX. Our results demonstrated that LASP1 was a pivotal modulator of the molecular characteristics of EMT by regulating vimentin in HBX-positive hepatoma cells. Furthermore, we found that vimentin also participates in cellular proliferation and migration mediated by LASP1 in HBX-associated hepatoma cells. These results suggested that HBX could promote the development of HCC via LASP1 to induce EMT that dependent on vimentin in hepatoma cells.

We also explored the molecular mechanisms associated with the upregulation of vimentin mediated by LASP1 in HBX-positive hepatoma cells. Previous researches show that multiple signal pathways, including PI3-K [29], ERK [30], and STAT3 [31], contribute to the expression of vimentin in different cells. However, whether these signal pathways are associated with the expressions of vimentin mediated by LASP1 in HBX-positive hepatoma cells is unknown. Consistent with our reported works [27], we found that HBX could induce the activation of these signal pathways. Furthermore, our results showed that PI3-K, ERK, and STAT3 pathways were responsible for vimentin expression mediated by LASP1 in HBX-positive hepatoma cells.

Besides these, current studies indicated that HBX could interact with different target proteins to protect them from ubiquitination and degradation [33, 34]. In the present study, we identified that HBX could directly interact with LASP1 and vimentin. To determine which region of HBX is capable of interacting with LASP1 and vimentin, three different HBX mutants were constructed, and we discovered that the N terminal of HBX with the OD domain could interact with LASP1, and the middle region of HBX with MT domain can bind to vimentin. In addition, our results were consistent with the findings from a previous study [25], which had shown the interaction of LASP1 and vimentin. Furthermore, our findings indicated that HBX could increase the levels of vimentin proteins by protecting it from ubiquitination mediated protein degradation. Besides, the effect of HBX on vimentin stability was dependent on the presence of LASP1 protein in hepatoma cells. Thus, these new findings revealed that multiple molecular mechanisms are involved in the increase of vimentin mediated by HBX via LASP1.

## Conclusion

In summary, the present study characterized the expression and associated molecular functions of vimentin mediated by HBX in HCC and identified LASP1 as a regular of vimentin protein expression in HBX-positive hepatoma cells. Mechanistically, LASP1 not only could promote multiple signal pathways to enhance vimentin expression but also bind to vimentin to increase its protein stability to facilitate the cellular proliferation and migration mediated by HBX. Therefore, our study provides a novel understanding of the role of vimentin and associated molecular mechanisms with LASP1 in HBX-mediated hepatocarcinogenesis and may provide new therapeutic targets for HBV-related HCC treatment.

## Data Availability

The data that support the findings of this study are available from the corresponding author upon reasonable request.

## Abbreviations

HBV: Hepatitis B virus
HBX: HBV X protein
EMT: Epithelial-mesenchymal transition
HCC: Hepatocellular carcinoma
LASP1: LIM and SH3 domain protein 1
CHX: Cycloheximide
IHC: Immunohistochemistry
Co-IP,: Co-immunoprecipitation
